# Analysis of Bacterial Community Characteristics, Abundance of Antibiotics and Antibiotic Resistance Genes Along a Pollution Gradient of Ba River in Xi’an, China

**DOI:** 10.3389/fmicb.2018.03191

**Published:** 2018-12-21

**Authors:** Yongjing Guan, Jia Jia, Lang Wu, Xue Xue, Guo Zhang, Zaizhao Wang

**Affiliations:** Shaanxi Key Laboratory of Molecular Biology for Agriculture, College of Animal Science and Technology, Northwest A&F University, Yangling, China

**Keywords:** antibiotics, antibiotic resistance genes, bacterial community, wastewater treatment plant, resident domestic sewage, urban river

## Abstract

The microbial communities in freshwater have raised concerns about the ecosystem and human health. Many ecological environmental problems have been found in urban river because of the unreasonable use and long-term wastewater discharge. In this study, we explored the bacterial community composition, abundance of 14 antibiotics and 21 antibiotic resistance genes (ARGs), and water environment features in seven water samples and seven sediment samples from Ba River in Xi’an, China. Results showed *Proteobacteria* and *Bacteroidetes* were the dominant phyla in all samples, and sediment samples had a higher bacterial diversity and richness than it in water. Bacterial communities of site 5 and 6 were clustered in discrepant patterns compared to those at remaining sites from other samples. It might be influenced by nutrients, heavy metals and antibiotics. Antibiotics concentrations ranged from 1.26 to 1.61 × 10^3^ ng L^-1^ in water samples and 1.55 to 4.05 × 10^2^ μg kg^-1^ in sediment samples. Sulfamerazine (SM1) and erythromycin (ERY) were the chief antibiotics in water samples, while the level of oxytetracycline (OTC) and cefazolin (CFZ) were higher in sediment samples. Canonical correspondence analysis showed that trimethoprim (TMP) was significantly related to *Acinetobacter* in W6, and that SM1 and OTC had positive correlation with *Arcobacter* in W5. The *tetC, bla_TEM_*_,_ *ermF* and *sul1* had higher pollution abundance ranging from 10^-4^ to 10^0^ copies/16S rRNA gene copies in all samples. Significant correlations were observed between ARGs and matching antibiotics, suggesting that antibiotics can pose the selective pressure on ARGs in this river. In summary, these finding might provide some new data to the limited information available on the bacterial community characteristics, abundance of antibiotics and ARGs in urban river of China.

## Introduction

As an important ingredient of the ecosystems for terrestrial freshwater and sediment, bacterial community play a crucial role in microbial food webs, biogeochemical cycles, energy flows and the decomposition of pollutants in the aquatic environment. As a result, bacterial community has raised concerns about the ecosystem and human health (Ruiz-González et al., 2015; Monard et al., 2016; Miao et al., 2017). The changes of bacterial community are reliable signals for pollution in water or sediment (Garrido et al., 2014; Su et al., 2017). Antibiotics, as an emerging contaminant in aquatic environments, have posed potential adverse effects on humans, animals and microorganisms (Milic et al., 2013; Petrie et al., 2015). In addition, the antibiotic-resistant bacteria and antibiotic-resistant genes (ARGs) in water increased the risks for aquatic ecological balance and human health (Michael-Kordatou et al., 2017; Zhu et al., 2017). Thus, it is indispensable to explore the bacterial community, antibiotics and ARGs in freshwater ecosystems.

As the major ingredient of freshwater ecosystems, urban river ecosystem is used for agricultural irrigation, entertainment, and it plays a key role in the source of water, traffic channel and pollution purification. However, with increasing human activities, urban rivers are polluted by wastewaters from communities, hospitals, pharmaceutical industries and animal husbandry. In previous studies, large amounts of antibiotics and high levels of ARGs have been identified in urban rivers (Sim et al., 2011; Rico et al., 2014; Rodriguez-Mozaz et al., 2015). Many reports have suggest that the abundance of ARGs may be correlated with heavy metal (He et al., 2017; Zhao et al., 2017). Besides, the structure of bacterial community was notably affected (Subirats et al., 2017). Pathogenic bacteria such as *Clostridium difficile, Arcobacter butzleri, Escherichia coli* and *Kluyvera georgiana* were detected in polluted rivers (Jia et al., 2017; Yang et al., 2017), which could cause potential health risks to residents nearby. Additionally, a recent study has proved that antibiotics can affect microbiological compositions in water (Xi et al., 2015). Therefore, it is worthwhile to explore the relationships among antibiotic, ARGs and bacterial community in urban river, which plays a major role in providing a stable and healthy environment for human beings and guaranteeing the city sustainable development (Zhou et al., 2017).

Ba River, one of the main water resources in Xi’an, China, has many ecological problems caused by unreasonable exploitation and long-term wastewater discharge. A survey of primary health care settings of Xi’an showed 40.5% was prescribed antibacterial in 780 outpatient prescriptions from April to May in 2013. And the rate of combined use was 8.2% (Ye et al., 2016). In 2015, the defined daily dose of antibacterial drugs was 4.83 × 10^6^ in 13 tertiary hospitals in Xi’an (Li and Hu, 2016). Our laboratory studies indicated that significant gradient pollution exist along Ba River, including high concentrations of phenolic and steroidal endocrine disrupting compounds in the middle and lower reaches, and antibiotic pollution in the downstream (Jia et al., 2018; Wang et al., 2018). So far, there are no studies on the bacterial community structure in response to the gradient pollution in river system. The previous studies on microbial composition or environmental quality mainly focused on water bodies (Abia et al., 2017). Therefore, in this study, we examined bacterial community, abundance of antibiotics and ARGs and environment features in both water and sediment samples from different sites along the pollution gradient.

## Materials and Methods

### Sampling Sites and Sample Collection

**FIGURE 1:**
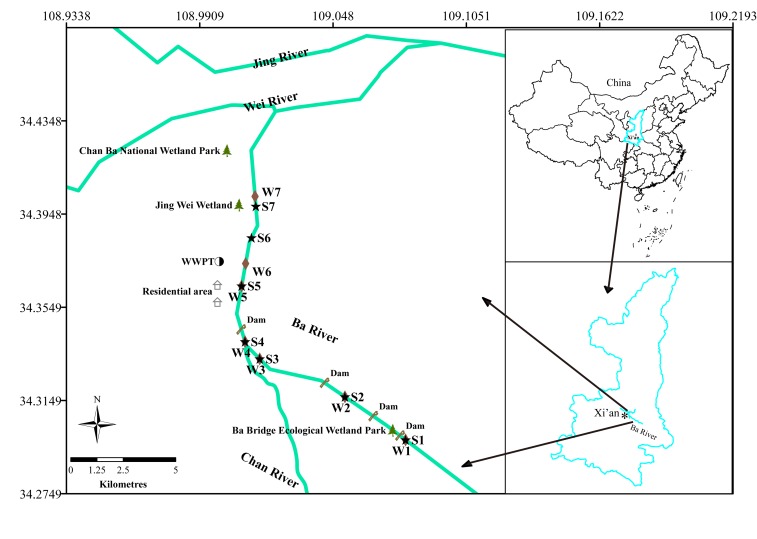
Map of the study area and the sampling sites.

In this study, surface water and sediment were sampled in May 2017 at 14 locations in triplicate along the Ba River in Xi’an (Figure 1). Seven of these samples, labeled as W1, W2, W3, W4, W5, W6, and W7, were collected from water. Sediment samples were marked with S1, S2, S3, S4, S5, S6, and S7. The sampling sites for W1 and S1 were located at the upper reach of the Ecological Wetland Park of Ba River Bridge. Sites for W2 and S2 were located in the downstream of the Riverside Park. Sites for W3 and S3 were located at the hundred meters upstream from W4. Sites for W4 and S4 were located at the mouth of Chan River, which flows into Ba River. The sites for W5 and S5 were located at a resident domestic sewage outlet. The site for W6 was located at the wastewater treatment plant (WWTP) discharge port. The site for S6 was located at the two hundred meters downstream from W6. The site for W7 was located close to Jing Wei Wetland, and S7 was located at several hundred meters upstream from Chan Ba National Wetland Park. Water samples (2.0 L) from the top 0.5 m of the water surface were collected. Sediment samples (50 g) were collected from the top 5 cm layer using a bottom sampler. All samples were collected using sterile containers and transported on ice to the laboratory for analysis.

### Antibiotics and Heavy Metal Concentrations and Water Environmental Features Analyses

Ultra-high performance liquid chromatography (UHPLC, Agilent 1290 Infinity, United States) coupled with tandem mass spectrometry (MS/MS, Agilent 6460 Triple Quadrupole, United States) and isotope dilution were used to determine the concentrations of fourteen antibiotics belonging to the sulfonamides, quinolones, tetracyclines, macrolides, β-lactams and amphenicols including sulfadiazine (SDZ), sulfamerazine (SM1), trimethoprim (TMP); norfloxacin (NOR), ciprofloxacin (CIP); oxytetracycline (OTC), tetracycline (TC), chlortetracycline (CTC); erythromycin (ERY), roxithromycin (ROX); cefotaxime (CTX), cefazolin (CFZ), penicillin G (PEN G); chloramphenicol (CHP). Trimethyl-^13^C_3_ caffeine was used as standards (Sigma-Aldrich Co., United States). Samples were pretreated and solid phase extracted according to the previous study (Luo et al., 2010). Chromatographic analyses were performed by injecting 2 μL extract into a ZORBAX Eclipse Plus C_18_ column (3.0 × 50 mm, 1.8 μm, Agilent Technologies, United States) at 35°C. The total mobile phase flow rate was 0.45 mL min^-1^. The washing gradient program of flowing phase was described in Supplementary Tables S1, S2 (CHP). The tandem MS analyses were performed in the positive electron spray ionization (ESI) and negative ESI (CHP ionization) modes in this study. The optimum MS conditions were: the source temperature, 325°C; dry gas temperature, 350°C; dry gas flow, 12 L min^-1^; nebulizer pressure, 45 psi; sheath gas heater temperature, 300°C; sheath gas flow, 6 L min^-1^; capillary voltage, 3000 V; Function, multiple reaction monitoring (MRM). Fourteen compounds and Trimethyl-^13^C_3_ caffeine used as standards (Sigma-Aldrich Co., United States) were described in Supplementary Table S3. The recovery rates varied between 63.4 and 107.9% (Supplementary Table S4).

Temperature (T), pH, specific conductivity (S), and dissolved oxygen (DO) levels of water samples were measured with the Multi 3410 Set KS1 (WTW, Germany). Water transparency was measured using a secchi disk (SD). The permanganate index [chemical oxygen demand (COD)] and the total contents of nitrogen (TN) and phosphate (TP) were measured according to the national standard method (Jia et al., 2018). The concentrations of six heavy metals (Cr, Cd, Cu, As, Pb, Hg) in water samples were analyzed by the inductively coupled plasma mass spectrometer (ICP-MS, Thermo Fisher Scientific, iCAP Q, United States). All measurements were conducted in triplicate.

### DNA Extraction

Water samples (1 L) were vacuum filtered through 0.22-μm filter. The filters were stored at -20°C before DNA extraction. Water DNA was extracted with the Water DNA kit (Omega Bio-tek, United States) according to the instructions of the manufacturer. For sediment, DNA from 0.5 g lyophilized samples were extracted with the Soil DNA kit (Omega Bio-tek, United States). The DNA quality was detected with 1% agarose gel electrophoresis, and the concentration was measured by a NanoDrop Spectrophotometer (Thermo Fisher Scientific Inc., United States). DNA samples were stored at -20°C for further analysis.

### Quantification of ARGs and 16S Ribosomal Ribonucleic Acid (16S rRNA)

The abundance of target genes were analyzed by qPCR including 16S rRNA and 21 antibiotic resistance genes (ARGs) including three sulfanilamide resistance genes (*sul1, sul2, sul3*), three quinolone resistance genes *(gyrA, qnrB, qnrS*), six tetracycline resistance genes (*tetA, tetB, tetC, tetM, tetW, tetZ*), three macrolide resistance genes (*ermB, ermC, ermF*), three β-lactam resistance genes *(bla_IMP4_, bla_NDM1_, bla_TEM_*), three amphenicol resistance genes (*cat1, cmlA, floR*). The 16S rRNA gene was quantified to normalize the abundance of the ARGs and to assess the total bacterial population. The primers used were designed in our laboratory (Supplementary Table S5; Pei et al., 2006; Xi et al., 2009; Jia et al., 2018). All qPCR assays were performed using CFX96TM Real-Time PCR Detection System (Bio-Rad, United States). The qPCR reactions were carried out in a final volume of 25 μL, using 1 × SYBR *Premix Ex Taq*^TM^, 0.4 μM of each gene specific primer, and 2.5 μL RT reaction solution. Each individual sample was run in triplicate using the following protocol: 95°C/30 s, 40 cycles of 95°C/5 s, 62°C/30 s. To ensure the specificity of each amplicon, a melting curve analysis was performed after amplification. CFX Manager software (Bio-Rad) was used to analyze the density of SYBR green I and to determine the threshold cycle (Ct) value. The qPCR efficiency (E) of each PCR reaction was calculated, and all the *E* values were between 90 and 110%. Plasmids containing ARGs and 16S rDNA were created to produce the standard curves as described previously (Pei et al., 2006). *R*^2^ values were more than 0.99 for all calibration curves.

### Amplification of 16S rRNA Genes and Sequencing

The V3-V4 regions within the 16S rRNA gene were amplified from the DNA extracts using the forward primer 341F (CCTACGGGNGGCWGCAG) and reverse primer 805R (GACTACHVGGGTATCTAATCC). Each primer was labeled with an Illumina adaptor sequence and a unique multiplex identifier code. The standard 50 μL polymerase chain reaction (PCR) system included 2x Phanta Max Master Mix (Vazyme, China), 10 μM forward and reverse primers, 5 μL of DNA template and 16 μL of ddH_2_O. Thermal cycling program of 3 min initial denaturation at 95°C, followed by 8 cycles at 95°C for 30 s, 55°C for 30 s, 72°C for 45 s, and a final elongation step at 72°C for 5 min. The amplicons were quantified using Quant-It Pico Green kit (Invitrogen, United States) with Qubit Spectrophotometer (Invitrogen, United States), and samples were pooled together for library preparation. The library concentration was measured with Agilent 2100 Bioanalyzer (Agilent Technologies, United States), and diluted to 4 nM using Tris pH 8.5. After denaturation, 6 pM of the combined sample library and PhiX control was loaded on a MiSeq Platform (Illumina; United States) using 600 cycles MiSeq Reagent Kit PE300 v3 (Illumina; United States), and 300 bps paired-end reads were cluster generated.

### Data Analysis

The sequence date generated in this study have been deposited in the NCBI Short Read Archive under accession number PRJNA504751. Raw FASTQ files were processed using the QIIME 1.80 (Caporaso et al., 2010), and the paired reads were joined with a combination of the FLASH 1.2.7 using the default setting and the PANDASEQ 2.9 (Magoc and Salzberg, 2011; Masella et al., 2012), and then joined sequences were quality filtered and analyzed with QIIME. The remaining sequences were chimeras detected and removed using UCHIME 4.2.40 (Edgar et al., 2011). Then they were clustered into operational taxonomic units (OTUs) using UPARSE 7.0, with 97% sequence similarity. Ultimately, they were identified down to different taxonomic levels using Ribosomal Database Project (RDP) classifier 2.2 at a bootstrap cutoff of 80% (Cole et al., 2014). A heat map was generated from the relative abundance of OTUs with R 2.15.3 to classify provincial patterns in bacterial community composition. Venn diagrams were performed using R 2.15.3. A phylogenic tree was generated from the filtered alignment using FASTTREE. Based on the OTUs information, rarefaction curves and alpha diversity referring to community diversity were also calculated by MOTHUR. The phylogenetic beta diversity, including principal coordinate analysis (PCA) and hierarchical clustering analysis was evaluated with Bray-Curtis distance created by QIIME.

The linear discriminant analysis (LDA) effect size (LEfSe) analysis was performed on the OTU table using the online Galaxy interface (Segata et al., 2011). LEfSe method uses the Kruskal-Wallis’s test to identify taxa with significant differences and performs LDA to evaluate the effect size of each feature. The LDA threshold score of 2.0 and a significant *p* of 0.05 were used to detect biomarkers. Canonical correspondence analysis (CCA) or redundancy analysis (RDA) was performed to find out the probable links among bacterial community structure, antibiotics/ARGs distribution and sampling sites, the associations among environmental parameters of water samples, bacterial community and sampling sites, and the correlations among antibiotics abundance, ARGs distribution and sampling sites using R software. In the analysis process, the significant variables were selection to examine the correction between these variables and bacterial community. Maps of sampling sites and geostatistical analysis were performed in ARCGIS 10.1. All the data were shown as mean ± standard error of the mean (SEM).

## Results

### Bacteria in the Surface Water and Sediment Samples

#### Diversity of the Bacteria Community

**FIGURE 2:**
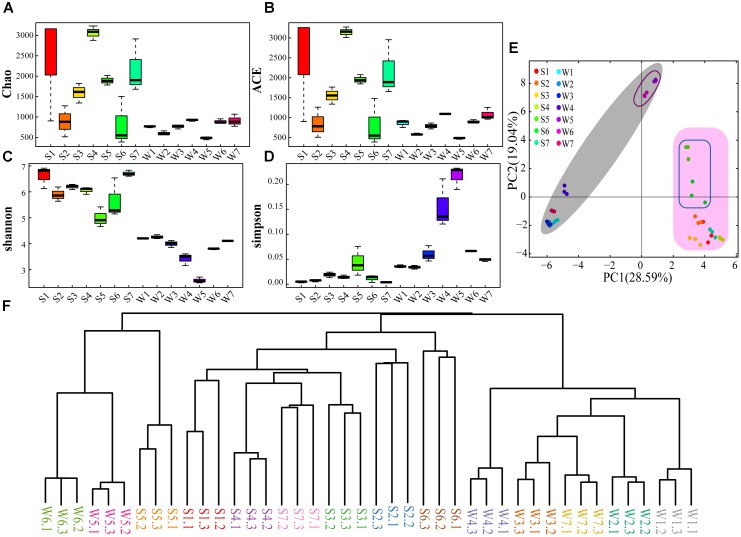
The diversity and phylogenetic structure of community composition. Sampling was performed at different sites for three times. W, Water samples; S, sediment samples. **(A)** Chao index. **(B)** ACE index. **(C)** Shannon index. **(D)** Simpson index. **(E)** PCA of the water and sediment samples based on the analysis of OTUs. **(F)** Bray-Curtis cluster tree of the water and sediment samples.

The sequence libraries from 42 samples contained 342 OTUs (smallest) in water samples and 2767 OTUs (largest) in sediment (Supplementary Table S6). The rarefaction curves of OTUs were saturated for all samples (Supplementary Figure S1), indicating that the sequencing depth was enough. Simultaneously, the rarefaction curves revealed that the community richness of sediment samples was higher than that of water samples. Chao and ACE indexes suggest that higher OTU richness was captured in sediment samples than water samples apart from site 6 (Figures 2A,B). This is also supported by Venn diagrams, demonstrating the water samples of the seven sites shared a lower number of OTUs compared with the sediment samples (Supplementary Figure S2). Shannon (species richness) and Simpson (species evenness) indexes illustrated diversity of bacterial community was higher in sediment sample than water sample (Figures 2C,D). Besides, the diversity index showed similar values for W1, W2, W3, W6, and W7 and lower values for W4 and W5. There was a clear difference between S5 and other sites of sediment samples (Figures 2C,D). The PCA showed that the bacterial community structure was significantly different between water and sediment samples (Figure 2E). Also, the bacterial community structures in site 5 and 6 were different from other sites (Figure 2E). These were also supported by the Bray-Curtis cluster tree of the water and sediment samples (Figure 2F).

#### Bacterial Community Composition

**FIGURE 3:**
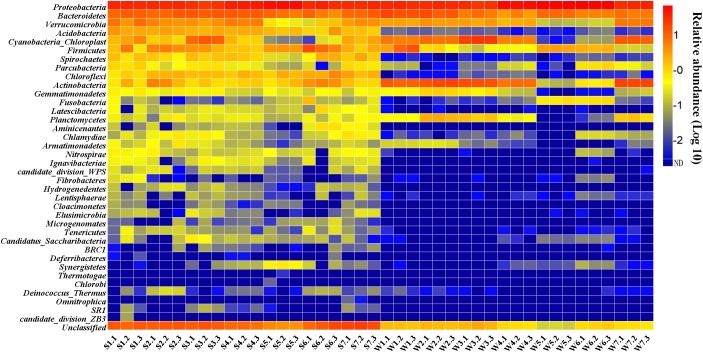
Percentage of different phyla in the water and sediment.

The 16S rRNA gene libraries detected 37 bacterial phyla of 42 samples. The community composition in the sediment and surface water samples is different (Figure 3). Two phyla (*Proteobacteria* and *Bacteroidetes*) had the high percentage in all water and sediment samples, accounting for 43.77–81.02% of the total reads in sediment and 42.23–95.09% in water using RDP Classifier at a confidence threshold of 80% (Supplementary Figure S3). In sediment samples, the two phyla were followed by a few other major (relative abundance > 1%) phyla, including *Verrucomicrobia, Acidobacteria, Firmicutes, Chloroflexi, Actinobacteria, Cyanobacteria* (except for S5 and S6), *Spirochaetes* (except for S5), *Parcubacteria* (only in S1, S3 and S7), *Chlamydiae* (only in S7), *Aminicenantes* and *Latescibacteria* (only in S6). However, *Acidobacteria, Chloroflexi, Spirochaetes, Chlamydiae* and *Aminicenantes* were minor abundance (<1%) in water samples. The two phyla were followed by *Actinobacteria* (except for W5 and W6), *Cyanobacteria* (except for W5 and W6), *Verrucomicrobia* (except for W5 and W6), *Firmicutes* (except for W3, W4, and W7), *Planctomycetes* (only in W2, W3, and W7), *Gemmatimonadetes* (only in W2), *Parcubacteria* (only in W6) and *Fusobacteria* (only in W5 and W6).

#### Significant Differences of Bacteria Communities in Surface Water and Sediment Samples

**FIGURE 4:**
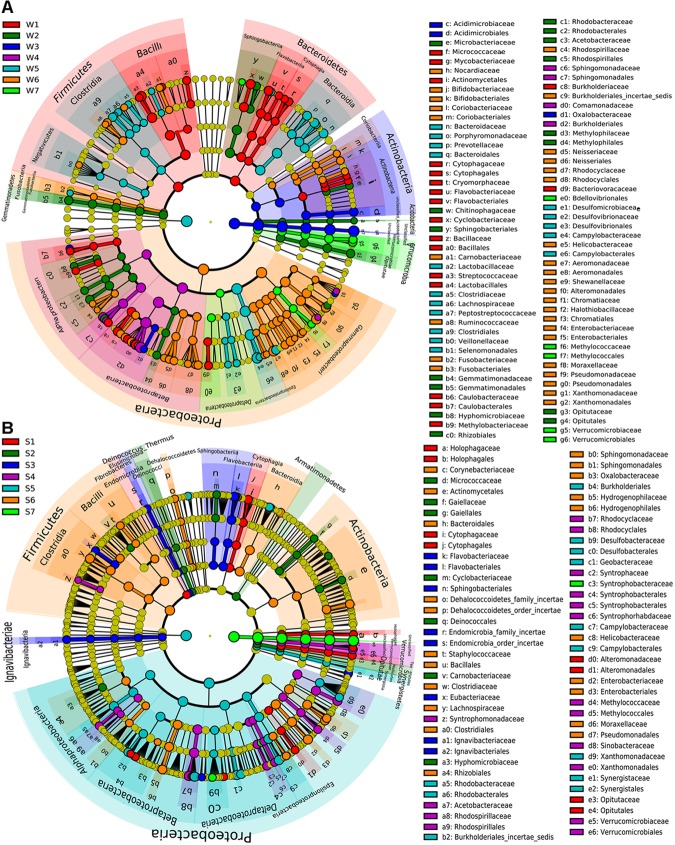
LEfSe analysis of bacteria abundance among different water samples **(A)** or sediment samples **(B)**. The phylum, class, order, family levels are listed in order from inside to outside of the cladogram and the labels for levels of order and family are abbreviated by a single letter. The red, green, blue, purple, light blue, orange and emerald green circles represent the bacteria enriched in the site 1, 2, 3, 4, 5, 6, and 7, respectively, whereas the yellow circles represent the taxa with no significant differences between 7 sites of the water **(A)** or sediment **(B)**.

The significant differences of specific bacteria in phylum, class, order and family levels among samples were shown in cladogram for LEfSe analysis (Figure 4). W1 was mainly enriched in *Firmicutes* and *Bacteroidetes* phyla. Also, *Lactobacillales, Bacillales, Flavobacteriales, Cytophagaceae, Coriobacteriaceae*, and *Caulobacterales* order were significantly enriched in W1 compared with other water samples (Figure 4A). Some bacterial taxonomy levels showed consistent abundance advantage from the phylum or order to genus in water samples, including W2 (*Gemmatimonadetes, Acidobacteria, Rhodobacterales, Methylophilales*, and *Opitutales*), W3 (*Acidimicrobiales*) and W7 (*Verrucomicrobiae* and *Methylococcales*, Figure 4A). Compared with other water samples, W5 was significantly enriched in *Clostridia, Bacteroidia*, and *Epsilonproteobacteria* (from class to family). Within the *Proteobacteria* phylum, *Alphaproteobacteria* and *Betaproteobacteria* (class) were significantly enriched in W4 while *Gammaproteobacteria* was mainly enriched in W6 (Figure 4A). Some bacterial taxonomy levels had consistent abundance advantage from phylum or class or order to genus in sediment samples, including S3 (*Lgnavibacteriae, Elusimicrobia*, and *Flavobacteriia*), S1 (*Opitutae, Holophagae*, and *Cytophagia*), S4 (*Verrucomicrobia, Methylococcales*, and *Rhodocyciaceae*), S6 (*Hydrogenophilales*, Figure 4B). S2 was mainly enriched in *Tenericutes, Armatimonadetes* and *Deinococcus-Thermus* (phylum), *Mollicutes* (class) and *Deinococcales* (order, Figure 4B). The four orders within *Proteobacteria* phylum and *Synergistetes* (from phylum to family) were significantly enriched in S5. *Firmicutes* (phylum) and *Clostridia* and *Bacilli* (class) showed abundance advantage in S6 (Figure 4B).

### Environmental Features of Water Samples and Their Relations to Bacteria

Concentrations of heavy metals, nutrients and physicochemical properties of water samples showed in Supplementary Figure S4. In general, SD, pH, Cd, and Hg had normal variations among all water samples. However, the abnormal high level of T, S, Cr, Cu, As, COD, TN, and TP and the low level of DO were found in W5 and W6, whose sampling sites are close to a resident domestic sewage outlet or a WWTP discharge port. CCA was performed to examine the potential relationships between environmental parameters and bacterial community composition. Forward selection was used to identify the most influential gradients, which represented the drivers of bacterial community composition changes. The significant environmental parameters (*p* < 0.05) were added to improve the model’s explanatory power. Results indicated that TP, TN, DO, SD, S, Cu, and Pb exhibited significant correlations with bacterial community. The biplot score showed that 63.38% of the variations were explained by CCA1 while 13.21% were explained by CCA2. In W4, *Betaproteobacteria* showed a positive association with SD and DO. In addition, *Epsilonproteobacteria* and *Bacteroidia* appeared to be linked with TN and Cu in W5, while *Gammaproteobacteria* was associated with TN and S in W6.

### Antibiotic Concentrations in the Water and Sediment Samples and Their Relations to Bacteria

**FIGURE 5:**
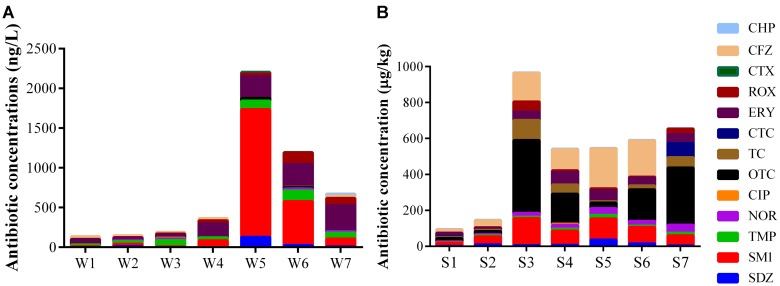
Concentration of antibiotics in water **(A)** and sediment **(B)** samples.

Fourteen antibiotics were detected at concentrations ranging from 1.26 to 1.61 × 10^3^ ng L^-1^ in water samples and 1.55 to 4.05 × 10^2^ μg kg^-1^ in sediment samples (Figure 5). SM1, TMP, ERY, and ROX were detected in all samples, while PEN G was not found for all samples. Besides, SDZ, NOR, OTC, TC, and CHP were found for all sediment samples. The relatively low concentrations of the total antibiotics were detected in site 1 and 2 including water and sediment samples, where are the upstream of the Ba River city section. The concentrations of all the detected antibiotics consistently increased for water samples from site 1 to 5. The high concentrations of antibiotics were found in W5, and they consistently decreased from site 5 to 7. In sediment samples, the total concentrations of antibiotics apart from S3 were increased continuously along the river flow, while S3 had the highest concentrations.

**FIGURE 6:**
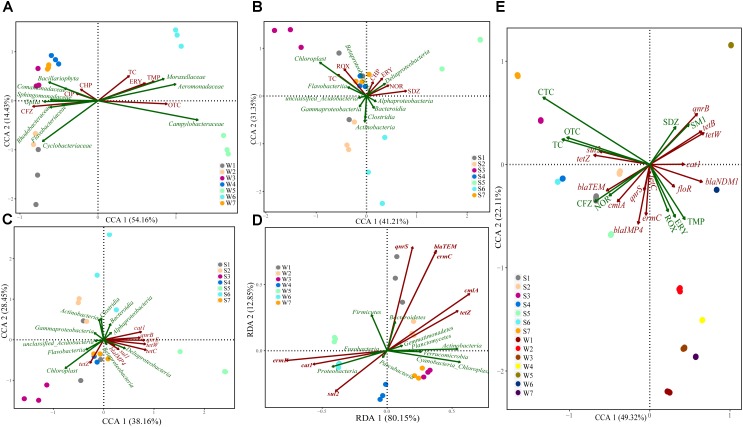
Canonical correspondence or Redundancy analysis of the relationship between bacterial communities and antibiotics/ARGs distribution **(A–D)**, and the correlations among antibiotics abundance and ARGs distribution **(E)**. Samples are performed at different sites for three times in different color solid circles separately.

Canonical correspondence analysis was performed to examine the potential relationships between antibiotics and bacterial community. Results showed that water bacterial community (family) was significantly shaped by several key antibiotics, including TC, ERY, TMP, OTC, CHP, CIP, and CFZ (Figure 6A). OTC and CFZ were kept separate from each other on the CCA1 (explaining 54.16% of the variations), and *Campylobacteraceae, Gplla, Sphingomonadaceae*, and *Rhodobacteraceae* were scattered on the CCA1 compared to other bacteria. In sediment samples (Figure 6B), the overall pattern of the bacterial community (class) was significantly related to TC, ROX, CHP, ERY, NOR, and SDZ. SDZ was positively correlated with the CCA1 (explaining 41.21% of the variations), whereas *Actimobacteria* was on the CCA2 (explaining 31.35% of the variations). *Cyanobacteria* showed a positive association with TC and ROX. In addition, *Deltaproteobacteria* appeared to be linked with ERY and NOR.

### Occurrence and Abundance of ARGs and Their Relations to Bacteria

**FIGURE 7:**
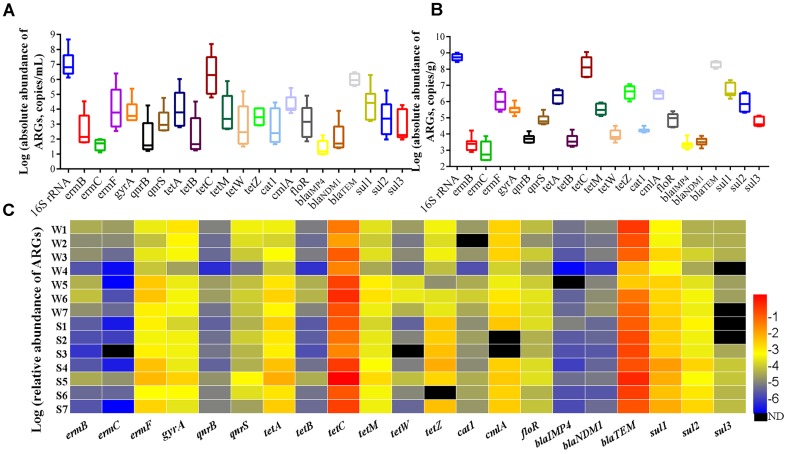
Absolute abundance of ARGs in water **(A)** and sediment **(B)** samples, and relative abundance of ARGs **(C)**.

The total bacteria load (16S rRNA gene copy numbers) were in the range of 1.34 × 10^6^ to 4.64 × 10^8^ copy numbers per mL (copies/mL) in water samples (Figure 7A) and 2.78 × 10^8^ to 1.03 × 10^9^ copy numbers per g (copies g^-1^) in sediment samples (Figure 7B), and the absolute abundance of ARGs in sediment was 1–3 orders of magnitudes higher than that in water, illustrating that the sediment was a natural reservoir of ARGs. Relative abundance of ARGs (the absolute abundance of ARGs normalized to the absolute abundance of 16S rRNA) in all samples was shown in Figure 7C. Overall, the *tetC, bla_TEM_, ermF, sul1, cmlA*, and *gyrA* were the predominant ARGs with the abundance ranged from 10^-4^ to 10^0^ copies/16S rRNA. The relative abundance of ARGs was not obviously different between the sites of upstream (site 1 and 2) and downstream. The six tetracycline resistance genes were detected in all samples (except *tetW* in S3 and *tetZ* in S6). The *tetC* had the highest relative abundance with values ranging from 2.23 × 10^-2^ to 9.92 × 10^-1^ in water and 4.99 × 10^-2^ to 4.09 × 10^0^ in sediment. For three β-lactam resistance genes, the *bla_TEM_* was the most abundant with values ranging from 5.14 × 10^-3^ to 6.68 × 10^-1^ in water and 1.99 × 10^-1^ to 1.16 × 10^0^ in sediment, while the *bla_IMP4_* was the lower abundance than other detected ARGs in water samples. Among the three macrolide resistance genes, the *ermF* had the highest relative abundance ranging from 1.26 × 10^-4^ to 9.38 × 10^-3^ in all samples, and it was higher in sediment than matching water samples. The relative abundance of three sulfanilamide resistance genes ranged from 4.12 × 10^-5^ to 2.47 × 10^-2^ in all samples, with higher abundance in sediment than in water.

CCA and RDA were performed to identify the potential relationships between the most significant ARGs and bacterial community structure, which were based on their relative abundance. RDA showed that the bacterial community (phylum) was significantly related to the dominant ARGs in water samples (Figure 6D), such as *cat1, ermF, sul2, qnrS, bla_TEM_, ermC, cmlA*, and *tetZ*, with 80.15% of the constrained variance explained by RDA1 and 12.85% by RDA2. In sediment samples, the variation, of which 38.16% could be explained by CCA1 and 28.45% by CCA2. CCA showed that the bacterial community (class) was significantly related to *cat1, qnrB, qnrS, tetW, tetC, sul1, bla_IMP4_* and *tetZ* (Figure 6C).

## Discussion

In our study, 16S rRNA sequencing techniques were employed to identify bacterial community structures from the Ba River. There was a clear differentiation of bacterial communities between sediment and surface water. For example, relative abundance of *Acidobacteria, Chloroflexi, Spirochaetes, Chlamydiae*, and *Aminicenantes* in sediment were higher than 1%, while those were minor abundance (<1%) in water samples. Additionally, the results showed that bacterial diversity and richness in sediment were relatively higher than those in water, suggesting that sediment in river environments may serve as a reservoir of microorganisms and a mainly environmental monitor. These results are consistent with previous studies demonstrating a higher concentration of microorganisms in sediment than that in water (Zeng et al., 2016).

Additionally, the bacterial communities in W5 and W6 were clustered away from the other water samples, and interestingly similar results were founded in S5 and S6. It should be noted that site 5 and 6 were close to domestic sewage outlet and WWTP discharge port. Thus, bacterial communities in site 5 and 6 might be affected by wastewater from residential area and WWTP. The previous findings also indicated that the sediment bacterial community in Tai Lake were affected by four different pollution sources (Wang et al., 2016). *Proteobacteria* and *Bacteroidetes* were proved to be the dominant components in this study. Similar results in sediment samples were also found in WWTPs in Colombia and China (Silva-Bedoya et al., 2016; Guo et al., 2017). Within the *Proteobacteria* phylum, *Alphaproteobacteria* and *Betaproteobacteria* class were significantly enriched in W4, whose sampling site is close to the confluence of Ba River and Chan River which is also received wastewater from WWTPs (Zhang et al., 2015). LEfse also showed that the four classes within *Proteobacteria* phylum were significantly enriched in S5 polluted by domestic sewage, which was consistent with previous investigation that *Proteobacteria* had the highest percentage in all river water samples contaminated by livestock breeding wastewater (Yang et al., 2017). In this study, *Bacteroidia* was significantly enriched from class to family in W5. These results suggest that *Proteobacteria* and *Bacteroidia* could survive in the wastewater environment, and their high abundance and diversity might be a reflection of human feces contamination. Some previous studies have also demonstrated that *Bacteroidales* order was enriched in the gut microbiota of many mammals and that specific species within this order have been proposed as fecal indicators (Fremaux et al., 2009; Liang et al., 2012). It was reported that *Bacilli* was the most active bacteria in the de novo DNA synthesis of the spore germination in dried river sediment, suggesting that *Bacilli* could become primary microbial colonizers and then passively release planktonic bacteria into the freshwater (Fazi et al., 2008). Besides, previous study indicated the wide distribution of *Clostridia* DNA in sediment samples more than surface water (Marcheggiani et al., 2008). Similarly, in the *Firmicutes* phylum, *Clostridia* and *Bacilli* class showed abundance advantage in S6 sediment sample in this study. The *Firmicutes* phylum was associated with wastewater samples with high antibiotics and extreme environmental conditions (Novo et al., 2013; Meng et al., 2016). Also, CCA showed that *Epsilonproteobacteria* and *Bacteroidia* were linked with TP, Cu, SM1, and OTC in W5, and *Gammaproteobacteria* were associated with TN, S, TMP, and TC in W6, which suggested that the changed abundance for these bacterial groups might result from both the antibiotics and the co-existing pollutants in water (Li et al., 2011). Based on the above, we speculate that the complicated impacts of the possible pollutants such as nutrients, heavy metals and antibiotics might prompt to select the appropriate bacterial groups and subsequently change the microbial diversity and composition.

Antibiotics analysis showed that high concentrations of SM1 and ERY were detected in water samples and high concentrations of OTC and CFZ were detected in sediment samples. The tetracyclines were mainly accumulated in sediment compared to water samples, whereas the concentration of TMP in the water was an order of magnitude higher than the corresponding sediment apart from site 4 and 7. The possible explanation was that the different distribution of antibiotics should be dependent on antibiotic physicochemical stability and geographical areas characteristics. Along the river flow from site 5 to 7, the concentration of OTC decreased in water samples and increased in sediment samples, supporting that tetracyclines were highly absorbed in organic matter, sediment and soil (Christou et al., 2017). The jump of ERY level at site 4 and its consistent increase in water samples from site 4 to 7 indicated that ERY was from Chan River, resident domestic sewage and the WWTP near by the river. There was no substantial increase in sediment ERY from site 4 to 7 with only somewhat elevation in S4 and S5. The shorter distance from the confluence of Chan River to the downstream rubber dam (less than 400 m) may facilitate the precipitation of suspended particles containing abundant ERY from Chan River, which could account for the relatively higher level of ERY in S4. The direct discharge of untreated wastewater at site 5 might prompt more ERY into sediment. In the present study, the high concentrations of SDZ and SM1 were detected in site 5, indicating its main source was residential domestic sewage. Compared to site 5, the concentrations of SDZ and SM1 were distinctly decreased in site 6 and 7, suggesting that the SDZ and SM1 might be removed by the WWTPs before their discharge into river near site 6. It might be attributed to the high removal efficiency for sulfonamides (up to 99.3%) in WWTPs (Zhang et al., 2017). CCA results showed that TMP was significantly related to *Acinetobacter* in W6 (Supplementary Figure S5). Although some species of *Acinetobacter* are environmental commensal bacteria, other species might cause serious nosocomial infections and community-acquired infections. Furthermore the treatment of *Acinetobacter* infections was difficult due to intrinsic resistance to multiple antimicrobial agents (Dijkshoorn et al., 2007; Clark et al., 2016). A previous study has also described that several *Arcobacter* carrying multidrug resistance was related to human and animal disease (Ferreira et al., 2016). *Arcobacter*, known as fecal contamination indicators, has gained visibility as opportunistic pathogens (Novo et al., 2013). In this study, SM1 and OTC were positively related to *Arcobacter* in W5. Therefore, it indicated that antibiotics posed selective pressure on some opportunistic pathogens, which was contributed to the increase of resistant prevalence and had adverse impact on water quality and human health. The concentrations of antibiotics including CHP, ERY, NOR, and SDZ were positively correlated with the abundance of *Deltaproteobacteria* and negatively with *Actinobacteria* and *Clostridia* among sediment samples. In addition, *Cyanobacteria* showed a positive association with TC and ROX. Thus, we speculated that different responses of bacterial communities to antibiotic pollution probably due to antibiotics pose selective pressure on indigenous bacterial communities. These were consistent with a previous study, in which showed that *Deltaproteobacteria, Bacilli, Clostridia*, and *Epsilonproteobacteria* might be specifically associated with antibiotic (PEN G and OTC, respectively) polluted rivers (Xiong et al., 2015).

To investigate how the bacterial composition changes under antibiotic selective pressure, the abundance of 21 ARGs were determined in water and sediment samples. In the present study, the *tetC, bla_TEM_*_,_ *ermF* and *sul1* had a higher relative abundance ranging from 10^-4^ to 10^0^ copies/16S rRNA gene copies in all samples. Previous studies have indicated that the relative abundance of ARGs were higher than 10^-4^ in contaminated sites (Graham et al., 2011). Therefore, it was reasonable to assume that our samples had relatively higher pollution levels of ARGs. The concentrations of antibiotics in freshwater play an important role in the maintenance and enrichment of ARGs and antibiotic resistance (Xiong et al., 2015). In this study, significant correlations were observed between several ARGs and corresponding antibiotics. For examples, the *tetC* and *bla_TEM_* showed a similar tendency for the residue of OTC and CFZ, suggesting that antibiotics might pose the selective pressure on ARGs. Whereas, the relative abundance of *sul2* was negatively correlated with SDZ and SM1 (Figure 6E), Huang et al. (2017) indicated that *sul*2 was significant correlated to *intI*1 in water and sediment samples, which meant that *sul*2 gene were associated with the mobile genetic elements (Cheng et al., 2013). Researchers also found that *sul2* and *sul3* located on plasmids are transferable and stable even in the absence of antibiotics (Suhartono et al., 2016). Thus, this negative correlation between *sul2* and residue of sulfonamides might be attributed to mobility of *sul2* located on mobile genetic elements. RDA showed that the relative abundance of *qnrS, bla_TEM_, ermC, cmlA*, and *tetZ* displayed significant positive correlations with *Bacteroidetes* and *Actinobacteria*, as well as *cat1, ermF*, and *sul2* showed significant positive correlations with *Proteobacteria* in water samples. Researchers have found that the transfer of ARGs between bacterial species occurred mainly among *Proteobacteria, Firmicutes, Bacteroidetes*, and *Actinobacteria*, and mobile ARGs were most enriched in *Proteobacteria* (Hu et al., 2016). Similarly, in sediment samples, CCA showed that *Deltaproteobacteria* was significantly related to *cat1, qnrB, qnrS, tetW, tetC, sul1*, and *bla_IMP4_*. Based on the above, it indicated that ARGs might transfer among bacterial community by MGEs in this riverine system.

## Conclusion

The present study aimed to analyze the respond of microbial composition to the gradient pollution in river system. Wastewater from residential area and WWTP discharge may have contributed to the differences among bacterial communities in this riverine system. Sediment in river environments may serve as a reservoir of microorganisms. SM1 and ERY were the predominant antibiotics in water samples while high concentrations of OTC and CFZ were detected in sediments. TMP was significantly related to *Acinetobacter* in W6, and SM1 and OTC had positive correlation with *Arcobacter* in W5. Some antibiotics posed selective pressure on some opportunistic pathogens or indigenous bacterial communities. The *tetC, bla_TEM_*_,_ *ermF* and *sul1* had the higher pollution abundance ranging from 10^-4^ to 10^0^ copies/16S rRNA gene copies in all samples. And significant correlations were observed between ARGs and matching antibiotics. These finding provide new data to the limited information available on the bacterial community characteristics, abundance of antibiotics and ARGs in urban river of China.

## Author Contributions

YG and ZW contributed conception and design of the study. LW and GZ organized the database. JJ and XX performed the statistical analysis. YG wrote the first draft of the manuscript. JJ and ZW wrote sections of the manuscript. All authors contributed to manuscript revision, read and approved the submitted version.

## Conflict of Interest Statement

The authors declare that the research was conducted in the absence of any commercial or financial relationships that could be construed as a potential conflict of interest.
