# Supplementary material for: Analysis of Bacterial Community Characteristics, Abundance of Antibiotics and Antibiotic Resistance Genes Along a Pollution Gradient of Ba River in Xi’an, China
Source: Front Microbiol. 2018 Dec 21;9:3191. doi: 10.3389/fmicb.2018.03191 (PMC6308138; doi:10.3389/fmicb.2018.03191)
Supplement: Supplementary file 1 [file Data_Sheet_1.PDF]

Supplementary material

Analysis of bacterial community characteristics, abundance of antibiotics and antibiotic resistance genes in water and sediment from Ba River in Xi'an, China

Yongjing Guan, Jia Jia, Lang Wu, Xue Xue, Guo Zhang, Zaizhao Wang*

College of Animal Science and Technology, Northwest A&F University, Shaanxi Key Laboratory of Molecuar Biology for Agriculture, Yangling, Shaanxi 712100, China

* Corresponding Author:

Zaizhao Wang, PhD

College of Animal Science and Technology

Northwest A & F University

22 Xinong Road

Yang ling, Shaanxi 712100 China

Tel: +86-29-87092139

Fax: +86-29-87092164

E-mail: zzwang@nwsuaf.edu.cn

Number of Figures: 5

Number of Tables: 6

Table S1 The washing gradient program of the flowing phase for 14 compunds

Total Time/ min	mobile phase A （0.1% formic acid in Milli-Q water）/%	mobile phase B （acetonitrile）/%
0.50	95.0	5.0
3.00	70.0	30.0
4.50	10.0	90.0
6.00	10.0	90.0
7.00	70.0	95.0
8.00	95.0	5.0
12.00	95.0	5.0

Table S2 The washing gradient program of the flowing phase for chloramphenicol

Total Time / min	mobile phase A（0.1% formic acid）/%	mobile phase B（acetonitrile）/%	mobile phase C（10mmol/L ammonium acetate）/%
2.00	25.0	70.0	5.0
3.00	25.0	70.0	5.0
3.01	70.0	25.0	5.0
7.50	70.0	25.0	5.0

Table S3 The detection conditions and retention time of 14 compunds and caffeine

Compound	Precursor ion (m/z)	Product ion (m/z)	Retion time (min)	Frag voltage (V)	Collision energy (V)	Polarity
ROX	837.5	158	6.29	170	38	Positive
ERY	734.5	158.1	6.31	170	30	Positive
CTC	479	444	5.44	120	18	Positive
OTC	461	426	4.50	120	18	Positive
CTX	456.1	396.1	4.74	92	10	Positive
CFZ	455.1	323.2	5.17	92	10	Positive
TC	445	410	4.72	120	18	Positive
CIP	332.2	288.3	4.56	108	18	Positive
NOR	320.3	276.3	4.33	108	14	Positive
TMP	291.1	230	4.22	120	22	Positive
SM1	265	156	4.20	120	14	Positive
SDZ	251	108	3.06	96	25	Positive
C13-caffeine	198.1	140.1	4.07	92	18	Positive
PNG E	335.1	160	6.01	96	5	Positive
CHP	320.9	256.9	2.34	106	10	Negative

Table S4 S/N, linear equation, correlation coefficients and dynamic of 14 antibiotics

Compound	S/N (2ppb)	linear equation	R^2	dynamic（ng/mL）	Recovery
SDZ	69.1	y=0.797x	0.999	1—1000	86.7±1.1
SM1	99.4	y=0.582x	0.999	1—1000	87.9±2.4
TMP	953.1	y=2.921x	0.994	1—200	107.9±7.1
NOR	19.1	y=0.918x	0.999	1—1000	86.2±1.4
OTC	19.5	y=1.076x	0.992	1—1000	70.6±5.5
CIP	23.3	y=1.095x	0.996	1—500	71.9±2.3
TC	23.4	y=1.310x	0.998	1—1000	70.3±1.5
CTX	20.3	y=0.336x	0.999	1—1000	80.5±1.2
CFZ	19	y=0.080x	0.999	1—1000	83.0±1.9
CTC	4.2	y=0.615x	0.999	1—1000	63.4±2.1
ERY	2860.3	y=2.318x	0.996	1—1000	69.3±1.0
ROX	281.7	y=3.418x	0.997	1—1000	87.1±1.8
PEN G	80.5	y=0.407x	0.996	1—1000	75.5±4.2
CHP	165.2	y=97.2x+0.339	0.999	1—1000	95.7±4.2

Table S5 Primer sequences and PCR conditions.

Gene name	Foreword primer	Reverse primer	Annealing temperature (℃)	Amplicon length (bp)	Reference
ermB	TAACGACGAAACTGGCTAAAATAAG	AACATCTGTGGTATGGCGGG	60	419	3
ermC	GGGGATTTTACCCTTGAATTAGT	TCTTTTAGCAAACCCGTATTCC	60	279	3
ermF	TCCTTATGGCATTACTTCCGAT	GGACCTACCTCATAGACAAGTTTCA	60	186	3
gyrA	AGCGACCTTGCGAGAGAAAT	GGAACCGAAGTTACCCTGACC	60	338	3
qnrB	GGTACAAATATGGCTCTGGCAC	CTTTCAGCATCGCACGACTAA	60	196	3
qnrS	TTGCCCATCAAGTGAGTAATCG	AGGATAAACAACAATACCCAGTGC	60	341	3
tetA	TCTACATCCTGCTTGCCTTC	CATAGATCGCCGTGAAGAGG	60	210	3
tetB	GCAGGAAGAATAGCCACTAAATG	CAAATCCAGCCATCCCAAA	60	314	3
tetC	CTTGAGAGCCTTCAACCCAG	ATGGTCGTCATCTACCTGCC	60	418	3
tetM	TTTATCTGTATCACCGCTTCCG	ACAATCCGTCACATTCCAACC	60	154	3
tetW	TGTTTCCGCTTTGCTGTCG	TCCCTGATTCCTTCAATGCCT	60	404	3
tetZ	CCTTCTCGACCAGGTCGG	ACCCACAGCGTGTCCGTC	60	207	3
cat1	CTGGAGTGAATACCACGACGAT	GGATTGGCTGAGACGAAAAAC	60	132	3
cmlA	TTGGTACGACAGCGAGCACA	AAACAAGGCACGCCGAGG	60	234	3
floR	TTTGTCGCTTTCCGTCTACTTC	CTGCCATCCCAAGAACTCG	60	195	3
sul1	CGCACCGGAAACATCGCTGCAC	TGAAGTTCCGCCGCAAGGCTCG	65	163	1
sul2	TCCGATGGAGGCCGGTATCTGG	CGGGAATGCCATCTGCCTTGAG	57.5	190	1
sul3	TCCGTTCAGCGAATTGGTGCAG	TTCGTTCACGCCTTTCACCAGC	61	127	1
bla_{IMP4}	TACCGCAGCAGAGCCTTTG	CGTGGGGATGGATTGAGAA	60	295	3
bla_{NDM1}	GGGGATTGCGACTTATGCC	CAGCCACCAAAAGCGATG	60	219	3
bla_{TEM}	GCKGCCAACTTACTTCTGACAACG	CTTTATCCGCCTCCATCCAGTCTA	60	247	2
16S rRNA	CGGTGAATACGTTCYCGG	GGWTACCTTGTTACGACTT	60	142	3

Table S6 Summary of sequence library, OTUs and diversity and richness estimates at 97% level of water and sediment samples.

sample	Tag	OTU	sobs	chao	ace	shannon	simpson
W1	22772 ±523	530 ±4	530.33 ±3.79	760.66 ±19.99	860.50 ±94.67	4.20 ±0.02	0.036 ±0.003
W2	21610 ±440	459 ±12	459.00 ±11.53	603.82 ±49.57	585.52 ±20.62	4.26 ±0.08	0.034 ±0.004
W3	22714 ±839	562 ±15	561.67 ±14.57	760.13 ±46.08	788.65 ±72.74	4.00 ±0.13	0.060 ±0.016
W4	20233 ±1363	618 ±22	618.33 ±22.19	922.26 ±22.89	1096.61±0.54	3.42 ±0.24	0.156 ±0.049
W5	22661 ±619	361 ±17	360.67 ±17.24	481.45 ±35.11	481.37 ±22.08	2.58 ±0.11	0.217 ±0.024
W6	20969 ±1131	656 ±29	656.33 ±28.94	892.30 ±55.09	903.10 ±39.39	3.80 ±0.03	0.067 ±0.000
W7	22491 ±568	650 ±53	650.00 ±53.03	907.67 ±150.62	1075.34±156.17	4.11 ±0.01	0.049 ±0.003
S1	21029 ±1353	2135±1077	2135.00±1077.38	2410.14±1303.49	2472.39±1361.83	6.62 ±0.43	0.004 ±0.002
S2	24660 ±1312	732 ±241	731.67 ±241.33	890.73 ±379.62	849.98 ±381.17	5.89 ±0.28	0.007 ±0.002
S3	22561 ±2082	1499±179	1499.33 ±178.51	1594.04 ±240.83	1552.70 ±213.77	6.20 ±0.11	0.019 ±0.006
S4	19500 ±608	2276 ±71	2276.00 ±71.14	3054.98 ±162.60	3140.23 ±123.70	6.06 ±0.14	0.014 ±0.004
S5	19857 ±1793	1431 ±33	1431.00 ±32.60	1895.36 ±116.08	1953.97 ±115.98	4.99 ±0.39	0.044 ±0.029
S6	23873 ±3182	786 ±579	786.33 ±578.73	815.09 ±603.87	805.15±588.8416	5.66 ±0.77	0.012 ±0.007
S7	20732 ±2130	2002±494	2002.00 ±493.84	2166.20 ±657.29	2166.31 ±691.62	6.71 ±0.12	0.004 ±0.000

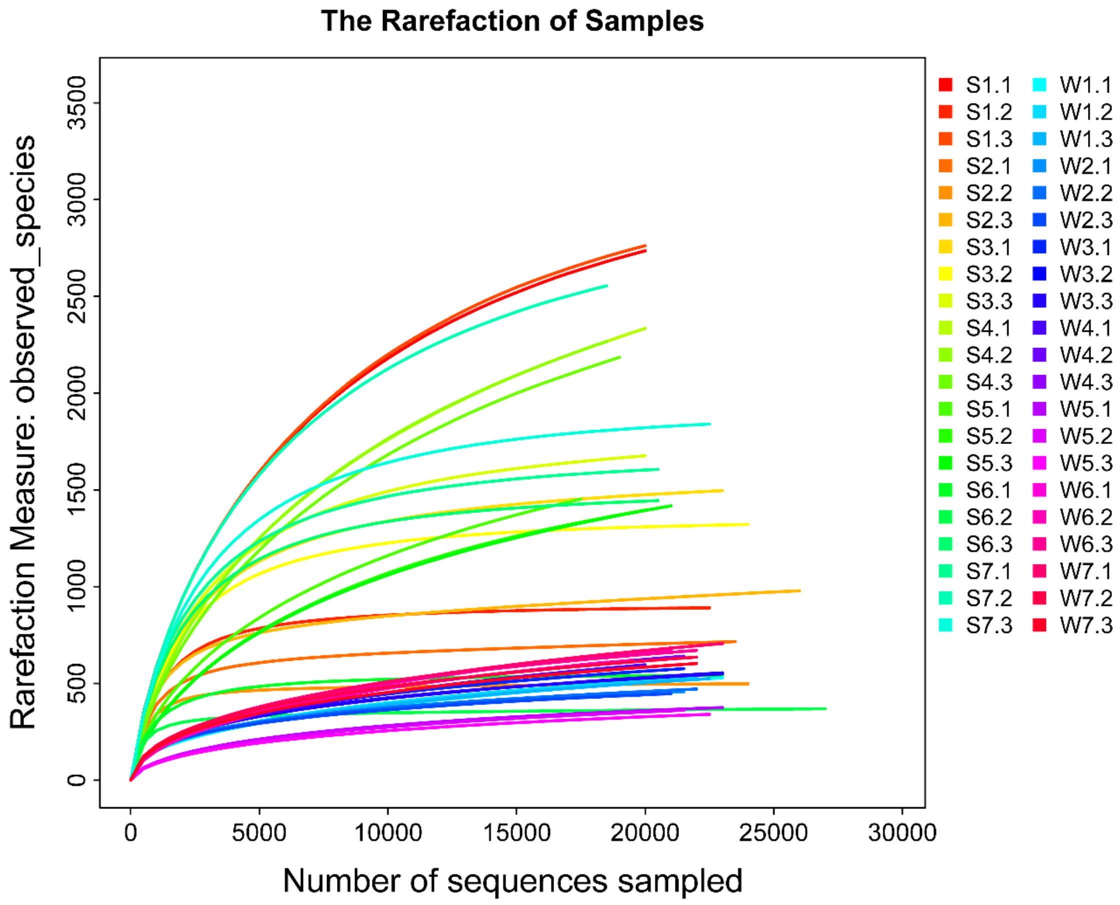

Fig. S1: Rarefaction curve of the water and sediment samples.

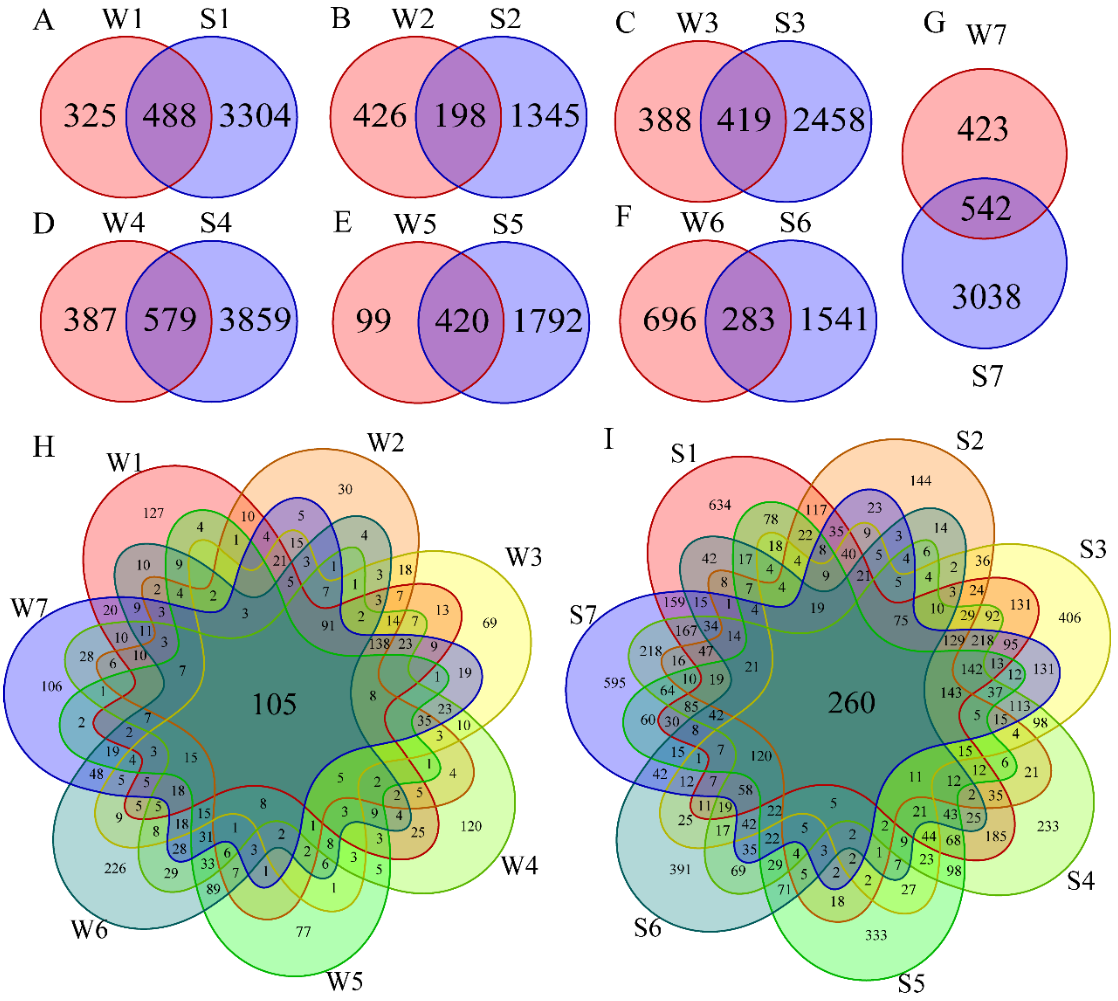

Fig. S2: Venn diagrams showing number of shared fraction and unique OTUs between water samples and sediment samples (A, B, C, D, E, F, G), among different water samples (H) and different sediment samples (I).

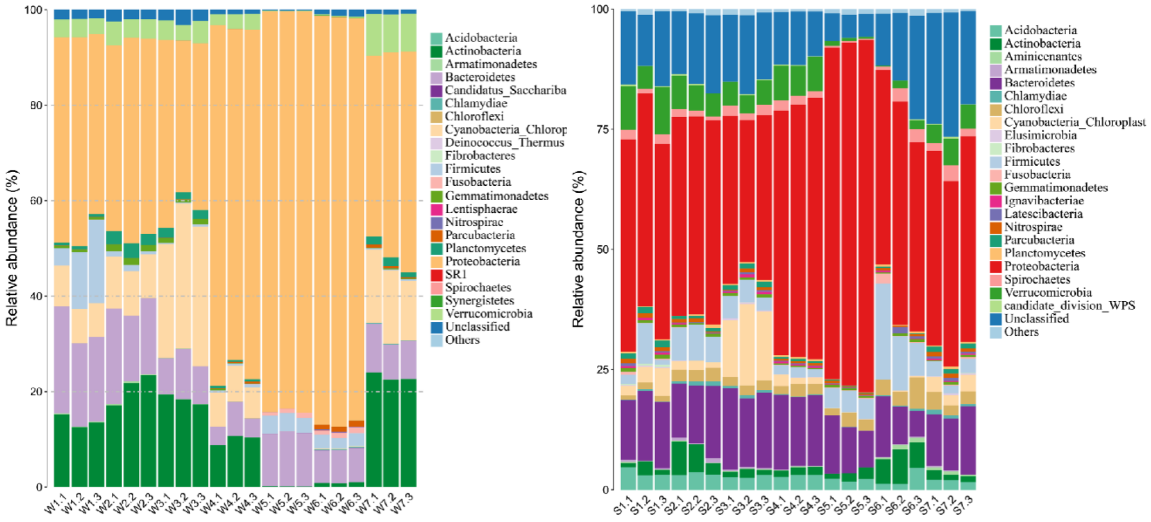

Fig. S3: Bacterial community composition of phyla (> 1%) in the water and sediment.

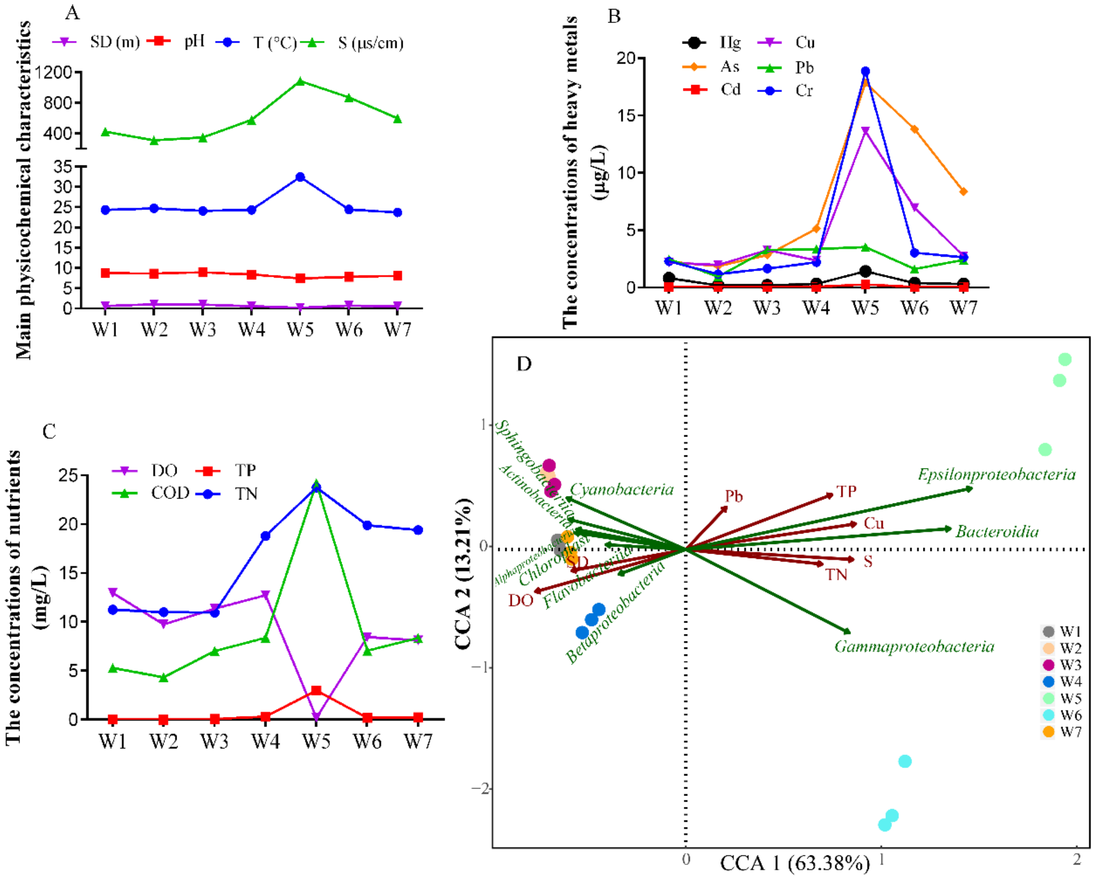

Fig. S4. Environmental parameters of water samples and relation to bacteria. Concentrations of physicochemical properties (A), heavy metals (B) and nutrients (C). Canonical correspondence analysis for the relationship between bacterial class and environmental parameters in seven water samples. Samples are performed at different sites (W1-W7) for three times in different colour solid circles separately (D). Cr, Cd, Pb, Cu, As, Hg were heavy metals. TP, total phosphate. TN, total nitrogen. SD, secchi disc. DO, dissolved oxygen. S, specific conductivity. T, temperature. COD, chemical oxygen demand.

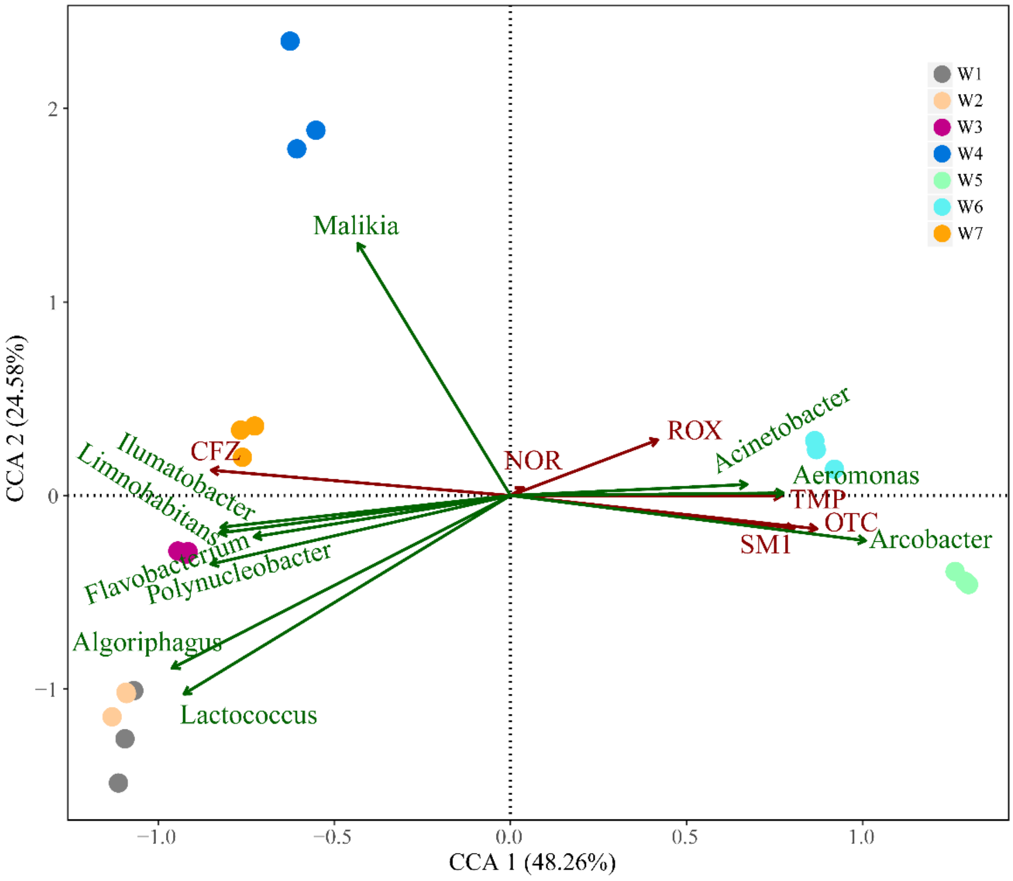

Fig. S5. Canonical correspondence analysis for the relationship between bacterial communities (genus) and antibiotics distribution. Samples are performed at different sites for three times in different colour solid circles separately.

Reference

1. Pei R, Kim SC, Carlson KH, Pruden A. Effect of river landscape on the sediment concentrations of antibiotics and corresponding antibiotic resistance genes (ARG). Water Res 2006; 40: 2427-35.

2. Xi C, Zhang Y, Marrs CF, Ye W, Simon C, Foxman B, et al. Prevalence of antibiotic resistance in drinking water treatment and distribution systems. Appl Environ Microbiol 2009; 75: 5714-8.

3. Jia J, Guan Y, Cheng M, Chen H, He J, Wang S, et al. Occurrence and distribution of antibiotics and antibiotic resistance genes in Ba River, China. Sci Total Environ 2018; 642: 1136-1144
